# Pathogenesis of Avian Bornavirus in Experimentally Infected Cockatiels

**DOI:** 10.3201/eid1802.111525

**Published:** 2012-02

**Authors:** Anne K. Piepenbring, Dirk Enderlein, Sibylle Herzog, Erhard F. Kaleta, Ursula Heffels-Redmann, Saskia Ressmeyer, Christiane Herden, Michael Lierz

**Affiliations:** Justus Liebig University Giessen, Giessen, Germany

**Keywords:** Avian bornavirus, proventricular dilatation disease, pathogenesis, cockatiels, psittacines, experimental infection, bornavirus disease, virus

## Abstract

Avian bornavirus (ABV) is the presumed causative agent of proventricular dilatation disease (PDD), a major fatal disease in psittacines. However, the influencing factors and pathogenesis of PDD are not known and natural ABV infection exhibits remarkable variability. We investigated the course of infection in 18 cockatiels that were intracerebrally and intravenously inoculated with ABV. A persistent ABV infection developed in all 18 cockatiels, but, as in natural infection, clinical disease patterns varied. Over 33 weeks, we simultaneously studied seroconversion, presence of viral RNA and antigens, infectious virus, histopathologic alterations, and clinical signs of infection in the ABV-infected birds. Our study results further confirm the etiologic role of ABV in the development of PDD, and they provide basis for further investigations of the pathogenetic mechanisms and disease-inducing factors for the development of PDD.

## Abstract 2

Inoculation induced persistent infection, clinical signs, and seroconversion.

Proventricular dilatation disease (PDD) is a significant cause of disease-related fatalities among birds, primarily psittacines (*1**–**3*). PDD has been observed in >50 psittacine species. Large parrots, including many endangered species, are the most frequently and most severely affected birds (*4*). PDD constitutes a threat to all parrot flocks and aviaries worldwide and endangers the protection and conservation of captive and wild psittacine species.

PPD is caused by a nonpurulent inflammation of the autonomic nervous system of the upper gastrointestinal tract, the peripheral and central nervous tissue, and the cardiac conduction system (*5**,**6*). Gastrointestinal and neurologic signs can appear alone or in combination (*4**,**7**,**8*). The clinical signs are nonspecific, and PDD can be definitively diagnosed only by pathohistologic detection of lymphoplasmacytic infiltrates of ganglia in the upper gastrointestinal tract. However, a negative finding cannot exclude the presence of PDD (*4**,**6**,**8**,**9*).

In 2008, 2 independent groups of research scientists described a new virus, avian bornavirus (ABV), which was amplified from samples from PDD-affected birds. Since then, 6 different ABV genotypes have been detected in psittacines birds. Additional genotypes have been detected in a canary (*Serinus canaria*), wild Canada geese (*Branta canadensis*), and trumpeter swans (*Cygnus buccinator*) (*3**,**10**–**12*). Recent studies substantiate the crucial role of ABV as the etiologic agent for PDD (*10**,**11*). Several scientific groups found ABV in 60%–100% of PDD-affected birds studied (*10**,**11**,**13**–**15*). Surveillance studies in aviaries showed that not all birds were affected after exposure to PDD-diseased and ABV-positive birds, and clinical signs and infection status varied considerably in birds that were affected. In addition, some ABV-positive birds showed no clinical signs (*16**–**20*). These facts indicate that host factors as well as features of the infectious agent, ABV, play a key role for disease induction (*18*).

Initial studies of experimental infections in birds fulfilled Henle-Koch postulates by using small numbers of animals. Gancz et al. (*21*) inoculated 3 cockatiels by multiple routes with brain homogenate containing ABV-4. Sixty-six days postinoculation (dpi), PDD-associated signs, including characteristic histopathologic lesions, developed in 2 of the 3 birds, and test results were positive for ABV-4. The implications of these findings were obscured by the fact that the brain homogenate also included sequences with partial analogy to viruses of the family *Astroviridae* and family *Retroviridae*. After inoculating 4-day-old mallards (*Anas platyrhynchos*; n = 15) with ABV-4, Gray and colleagues (*22*) detected ABV RNA in the feces and antibodies against ABV in serum, but they did not detect any clinical signs or PDD-associated lesions. Later, Gray et al. (*23*) inoculated 2 adult Patagonian conures (*Cyanoliseus patagonis*) with ABV-4. The conures were known to be chronic carriers of psittacine herpesvirus, but they appeared to be healthy. Antibodies against ABV were detected 33 dpi, and shedding of viral RNA was detected 62 dpi. Clinical PDD signs developed in both birds, after which 1 bird died and the other was euthanized. Histopathologic analysis showed typical PDD lesions. It was not determined whether the herpesvirus infection was a potentiating factor (*21**–**23*).

Reliable studies that include sufficient numbers of animals to address the host variability over an adequate investigation period are needed to clarify the pathogenetic effects of ABV infection on the development of PDD. More precisely, this implies the need for investigating the course of clinical signs, seroconversion, histopathologic lesions, and virus shedding and distribution in the tissues of affected birds. To further the understanding of ABV and the associated disease, we infected 18 cockatiels with ABV by using 2 different inoculation routes and monitored them for 33 weeks. Our findings show that a persistent ABV infection was induced by in all 18 birds and that it was possible to reliably reproduce all of the known natural ABV disease patterns.

## Material and Methods

### Inoculum and Sequencing

The virus for inoculation was originally isolated from the brain of a scarlet macaw (*Ara macao*) that died from PDD; the inoculation was passaged 6 times in the quail cell line CEC-32 (*24*). The persistently ABV-infected CEC-32 cells were suspended in medium with 2% fetal bovine serum, sonicated, clarified by centrifugation at 3,000 × *g* for 10 min, and assayed to determine infectivity. For inoculations, we used an infectivity titer of 10^4^ 50% infectious dose/mL.

By using the RNeasy Mini Kit (QIAGEN, Hilden, Germany) according to the manufacturer’s instructions, we isolated total RNA for sequencing from 200 μL of virus-containing supernatant. Total RNA was reverse transcribed by using random hexamer primers. PCR for parts of the large viral polymerase and the nucleocapsid protein genes of avian bornavirus was performed as described (*11*). We analyzed PCR products by using gel electrophoresis and purified the products for sequencing by using the QIAquick PCR Purification Kit (QIAGEN). Sequencing was carried out by LGC Genomics (Berlin), and BlastN (http://blast.ncbi.nlm.nih.gov/Blast.cgi) was used to align generated sequence information.

### Experimental Infection of Cockatiels

For inoculation purposes, we divided the cockatiels into 2 groups (groups ic and iv), each consisting of 9 animals. We intracerebrally (IC) inoculated birds in group ic and intravenously (IV) inoculated birds in group iv with 0.1 mL of the inoculum described above. All birds were under isoflurane anesthesia when inoculated. One bird remained untreated and served as a sentinel bird in group ic. Another group of 9 birds from the same flock served as controls; they remained untreated and were kept separate from the inoculated birds during the investigation period.

### Study Design (Sampling, Clinical Investigations, and Necropsies)

Over a period of 230 days, we surveyed the birds daily to determine their health status. We obtained swab samples from the crop and cloaca to test for the presence of ABV RNA by using real-time reverse transcription PCR as described (*10*). Cycle thresholds (C_t_) >36.0 were considered negative (*10*). Swab samples were obtained every other day until all birds of the respective group had ABV-positive test results, then specimens were obtained weekly. In parallel, once a week we obtained 0.3-mL blood samples for indirect immunofluorescence assay (IIFA) detection of antibodies against ABV, as described (*25*). For humane reasons, we euthanized birds with clinical signs typical of PDD (emaciation, undigested seed in the feces, neurologic signs) and reduced general condition. All remaining birds, including control birds, were euthanized 115 or 116 dpi or at the end of the trial (229 or 230 dpi).

We obtained samples of brain, eye, spinal cord, ischiadic nerve, adrenal gland, heart, liver, kidney, spleen, pancreas, crop, proventriculus, gizzard, intestine, pectoral muscle, and skin with feathers from all birds that died or were euthanized. For histopathologic analysis immunohistochemical testing, and other immunohistologic procedures, samples were fixed in 5% buffered formalin, embedded in paraffin wax, and used for preparation of 5-μm sections stained with hematoxylin and eosin. Samples from similar organs were frozen fresh for subsequent real-time PCR to detect ABV RNA. In addition, infectious virus was isolated by using samples from brain and retina as described (*25*). To exclude the presence of any other infection, we examined samples of blood, liver, and lung for bacteria; we used mycological staining to examine samples of proventriculus for infection with yeast; and we examined samples from all intestinal parts for parasites.

### Indirect Immunofluorescence Assay

Antibodies against ABV were detected by use of an IIFA on persistently Borna disease virus (BDV)–infected Madin-Darby canine kidney cells. For the assay, we used a 1:50 dilution of fluorescein isothiocyanate–conjugated goat anti-bird IgG (Bethyl Laboratories, Inc., Montgomery, TX, USA), as described (*25*).

### Immunohistochemical Testing

We performed immunohistochemical testing according to the avidin-biotin complex method, as described (*25**,**26*). This method uses a polyclonal rabbit antibody directed against the phosphoprotein and the X protein of BDV.

### Virus Isolation

For virus isolation, we performed infectivity assays as described by Narayan et al. (*27*) and Herzog et al. (*25*). Organ samples were homogenized, and 10-fold dilutions were prepared in GIBCO Glasgow Minimum Essential Medium BHK-21 1× (Invitrogen, Paisley, UK) with 10% fetal bovine serum. The mixture was then mixed with equal volumes of freshly dispersed cells of the quail cell line CEC-32 and then incubated on Lab-Tek Chamber Slides (Nunc, Roskilde, Denmark) for 6 days at 37°C. Virus replication was demonstrated by indirect immunofluorescence by using polyclonal serum specimens, which cross-reacted reliably with ABV antigen, from rats experimentally infected with BDV (*25*).

### Statistical Analysis

We used the Wilcoxon-Mann-Whitney-test to ascertain differences between the IC- and IV-inoculated groups. U <11 (critical value with α = 0.005) was considered highly significant for calculated test statistics.

## Results

### Sequence Analysis

We used BlastN (http://blast.ncbi.nlm.nih.gov/Blast.cgi) to compare newly generated sequences of parts of the nucleocapsid protein and large viral polymerase genes with sequences available from GenBank. Both genes showed highest accordance (99%) with ABV genotype 4 (EU781959.1 and FJ002323.1).

### Clinical Observations

During the 230-day investigation period, 5 of the 18 inoculated birds (3 from group iv and 2 from group ic) showed clinical signs typical of PDD: birds iv1 and iv3 had gastrointestinal signs, birds ic1 and iv5 showed neurologic signs, and bird ic2 had gastrointestinal and neurologic signs. In bird ic2, the following signs developed 33, 37, and 41 dpi, respectively: general symptoms, e.g., apathy; undigested seeds in the feces; and epileptic-like seizures. In birds iv3 and iv2, gastrointestinal signs first became obvious 116 and 126 dpi, respectively. In birds iv5 and ic1, which were only affected neurologically, signs were first noticed 159 and 199 dpi, respectively.

In addition, birds ic6 and iv2 died suddenly and unexpectedly, without obvious signs, 66 and 120 dpi, respectively. Three apparently healthy birds from each group (birds iv3, iv4, iv7, ic3, ic4, and ic8) were euthanized 115 or 116 dpi. At the end of the investigation period, 229 or 230 dpi, the remaining birds (iv1, iv5, iv8, iv9, ic5, ic7, and ic9) and the sentinel bird (se1), which all appeared clinically healthy, were euthanized (Table 1). The control birds were in a good health status during the entire investigation period and were euthanized with the other birds after 230 days.

**Table 1:** Premortem findings in cockatiels experimentally infected with ABV*

Bird	Age at inoculation,d/sex	Antibodies against ABV		RNA‡, first detected, dpi	Gastrointestinal signs			Neurologic signs		Died, dpi	Euthanized, dpi
		First detected, dpi	Titer†		First detected, dpi	Titer†		First detected, dpi	Titer†		
ic1	137/F	43	160	27	–	–		199	5,120	–	206
ic2	121/F	29	20	21	37	320		41	320	–	60
ic3	137/F	35	320	25	–	–		–	–	–	115
ic4	137/F	7	20	19	–	–		–	–	–	115
ic5	121/F	57	320	29	–	–		–	–	–	230
ic6	137/M	43	1,280	25	–	–		–	–	66	–
ic7	137/M	63	160	29	–	–		–	–	–	230
ic8	137/F	43	640	27	–	–		–	–	–	115
ic9	44/M	7	10	25	–	–		–	–	–	230
iv1	137/F	57	320	71	126	5,120		–	–	–	229
iv2	137/F	35	160	35	–			–	–	120	–
iv3	137/M	57	640	43	116	10,240		–	–	–	116
iv4	137/M	29	40	63	–	–		–	–	–	116
iv5	121/F	35	640	72	–	–		159	5,120	–	229
iv6	121/F	43	640	33	–	–		–	–	–	229
iv7	137/M	35	40	35	–	–		–	–	–	116
iv8	137/M	35	40	43	–	–		–	–	–	229
iv9	44/F	29	40	25	–	–		–	–	–	229
se1	137/M	NA	–	76	–	–		–	–	–	230

*Investigation period was 230 d. ABV, avian bornavirus; dpi, days postinoculation; ic, intracerebrally inoculated; –, not detected or not applicable; iv, intravenously inoculated; se, sentinel. †Antibodies detected by use of indirect immunofluorescence assay; titers <10.0 are considered negative. ‡Avian bornavirus RNA detected by real-time reverse transcription PCR as described by Honkavuori et al. (*10*); cycle thresholds >36.0 are negative.

### Gross Findings and Histopathologic Lesions

Necropsy revealed a dilated proventriculus in 7 of the 18 inoculated birds (ic1, ic2, ic3, iv1, iv2, iv3, and iv4), 4 of which had shown signs typical of PDD (Table 2). Dilatation of the proventriculus was most severe in bird ic2, which had both gastrointestinal and neurologic signs. No macroscopic alterations were detected in the remaining 11 inoculated birds, the sentinel bird, or the 9 control birds.

**Table 2:** Postmortem findings in cockatiels experimentally infected with ABV*

Bird	Died, dpi	Euthanized, dpi	Antibody titer†	Dilatation of proventriculus‡	Antigen detection§		Infectious virus¶
					p14	p24	
ic1			10,240	2	+	+	+
ic2	–	206	5,120	3	+	+	+
ic3	–	60	20,480	1	+	+	+
ic4	–	115	20,480	0	+	+	+
ic5	–	115	20,480	0	+	+	+
ic6	–	230	5,120	0	+	+	+
ic7	66	–	1,0240	0	+	+	+
ic8	–	230	1,0240	0	+	+	+
ic9	–	115	5,120	0	+	+	+
iv1	–	230	10,240	2	+	+	+
iv2	–	229	5,120	2	+	+	+
iv3	120	–	10,240	2	+	−	+
iv4	–	116	5,120	1	+	+	+
iv5	–	116	20,480	0	+	+	+
iv6	–	229	10,240	0	+	+	+
iv7	–	229	10,240	0	+	+	+
iv8	–	116	5,120	0	+	+	+
iv9	–	229	20,480	0	+	+	+
se1	–	229	<10	0	−	−	−

*Proventricular dilatation disease was confirmed in all birds by histopathologic confirmation of lymphoplasmacytic infiltrates of/near ganglia in the central nervous system and/or upper gastrointestinal tract. ABV, avian bornavirus; dpi, days postinoculation; ic, intracerebrally inoculated; +, positive; −, negative; iv, intravenously inoculated; se, sentinel. †Antibodies against avian bornavirus detected by use of indirect immunofluorescence assay; titers <10.0 are considered negative. ‡0, no dilatation; 1, mild dilatation; 2, moderate dilatation; 3, severe dilatation. §Detection of the X-protein (p14) and the phosphoprotein (p24) of Borna disease virus by immunohistochemical testing. ¶Re-isolation of infectious ABV in quail cell line CEC-32.

Histopathologic examination revealed that all infected birds and the sentinel bird had mononuclear infiltrates characteristic of PDD in a wide range of organs and of considerable severity. Most of the immune cell infiltrates were in the central nervous system and the gastrointestinal tract, but they were also present in heart; liver; kidney; pancreas; skin with feathers; and, in 1 case, the spleen. In some of the animals, the infiltrates had a follicle-like appearance. Infiltrates were not detected in the pectoral muscle of any birds. No other infections were detected during bacteriologic, mycologic, and parasitologic examinations. The control group did not have histopathologic alterations in organs and did not have any other infection.

### Detection of ABV RNA and Antibody against ABV

**Figure 1:**
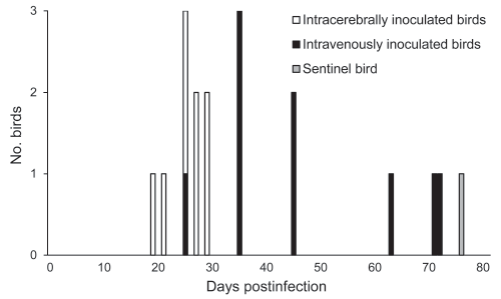
Timing of the first detection of avian bornavirus (ABV) RNA in cockatiels that had been intracerebrally or intravenously inoculated with ABV. ABV RNA was amplified significantly earlier in samples from intracerebrally inoculated birds compared with intravenously inoculated birds (α = 0.005 by using the Wilcoxon-Mann-Whitney test). A noninoculated sentinel bird, which was housed with the intracerebrally inoculated group of cockatiels, was the last bird to shed ABV RNA.

We detected ABV RNA in swab specimens from all inoculated birds and the sentinel bird. We obtained the first positive test results for group ic 19–29 dpi, whereas we first amplified ABV RNA from group iv samples 25–72 dpi (Figure 1). By using the Wilcoxon-Mann-Whitney test, we determined that ABV RNA was detected significantly earlier in group ic than in group iv (calculated value for test statistic U = 5.5, which is <11 [critical value with n_1_ = 9 and n_2_ = 9] and therefore considered to be highly significant, with α = 0.005). We obtained the first positive ABV RNA test results for the sentinel bird 76 dpi (Figure 1). The C_t_ for all birds constantly decreased during the trial, from 35.6 at first ABV RNA detection to 19.8 at last sampling (geometric mean values).

**Figure 2:**
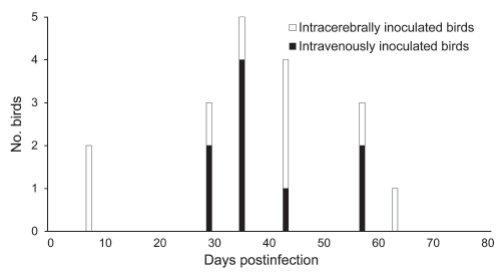
Timing of the first detection of antibodies against avian bornavirus (ABV) in cockatiels that had been intracerebrally or intravenously inoculated with ABV. The time of ABV antibody detection did not differ substantially between the 2 inoculation groups.

**Figure 3:**
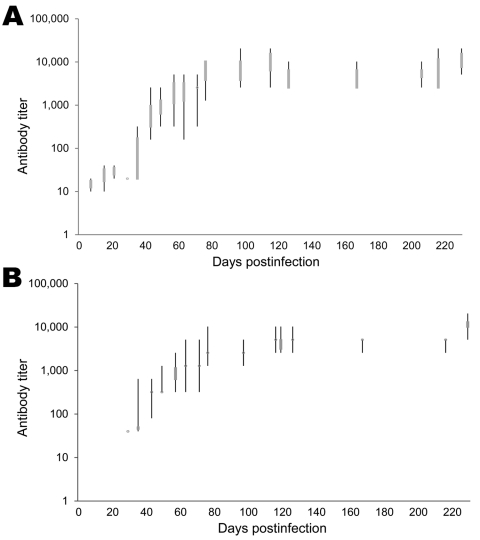
Avian bornavirus antibody in cockatiels inoculated intracerebrally (A) and intravenously (B) with avian bornavirus. In both groups, an exponential rise in antibody titers was detected within the first 12 weeks after inoculation and was followed by a plateau of high antibody titers (<20,480).

Antibodies against ABV were first detected in group ic 7–63 dpi, and group iv birds seroconverted 29–57 dpi (Figure 2). Statistical analysis did not show any noticeable difference between the ic and iv group (calculated value for test statistic U = 39.5, which is >21 [critical value with n_1_ = 9 and n_2_ = 9] and thus considered not significant, with α = 0.05). Antibody titers in groups ic and iv steadily increased to <20,480 during the investigation period (Figure 3). We did not detect ABV RNA or antibodies against ABV in control group birds during the investigation period.

### Detection of ABV RNA in Organs

**Figure 4:**
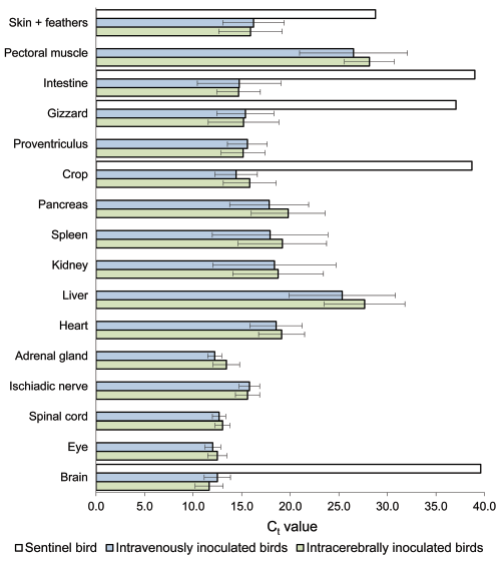
Detection of avian bornavirus (ABV) RNA in different tissues from cockatiels that had been intracerebrally or intravenously inoculated with ABV and from a noninoculated sentinel bird. The geometric mean cycle thresholds (C_t_) are shown with their respective standard deviations. C_t_ >36.0 is considered negative. Low C_t_, implying high amounts of ABV RNA, was detected in neuronal and gastrointestinal tissue.

We detected ABV RNA in all organs that were examined 115 or 116 dpi and 229 or 230 dpi. A high amount of ABV RNA (geometric mean C_t_ 12.0–17.0) was found in the central nervous system, gastrointestinal tract, and in skin with feathers. Moderate amounts of ABV RNA (geometric mean C_t_ 17.0–23.0) were found in heart, kidney, spleen, and pancreas. The lowest amounts of ABV RNA (geometric mean C_t_ 23.0–29.0) were found in pectoral muscle and liver. In the sentinel bird, only skin with feathers had positive test results (C_t_ 28.77), but brain, crop, gizzard, and intestine had high C_t_ (>36.0); all other organs remained negative for ABV RNA (Figure 4). We did not detect ABV RNA in the organs of control birds.

### Immunohistochemical Testing

The detection of viral phosphoprotein and X-protein was mostly restricted to the central nervous system and the heart. In some cases, ABV antigen was also found in some parts of the gastrointestinal tract. We detected X-protein in all group ic birds but not in the sentinel bird. We could not detect phosphoprotein p24 in bird iv3 or in the sentinel bird. ABV antigen was not found in any of the organs of the control birds.

### Virus Isolation

We isolated infectious ABV from all inoculated birds. No infectious virus was detected from the organs of the sentinel bird or birds in the control group.

## Discussion

PPD was first described in the late 1970s, but its etiology remained unknown until 2008 when ABV and its correlation to PDD were discovered. Numerous studies using naturally infected animals followed this discovery and substantiated the close association between ABV infection and PDD. This association has been further confirmed by several experimental trials (*21**–**23**,**28*) that used various bird species. However, detailed information about the occurrence of clinical signs, seroconversion, histologic lesions, viral RNA, and infectious virus using a statistically adequate group and sample size was still lacking. We reliably and successfully demonstrated these details in our study. We reproduced the natural pattern of ABV disease variation and the typical signs. We also achieved an infection rate of 100%, with the development of a persistent ABV infection in all animals, as indicated by the constant presence of ABV RNA, ABV antigen, infectious virus, and serum antibodies. These results are in agreement with other experimental data (*21*) and with infection in birds with natural PDD (*18**–**20**,**28*).

In our experiments, the discrepancies in infection status and in the development of clinical signs in infected but clinically healthy birds could be a result of several host factors (e.g., age, immune status), virulence, and adaption of the inoculated ABV suspension to the cell culture. These variations have already been described for mammalian BDV infections (*29**–**32*). The cockatiels most likely had considerable variability in their genetic makeup, in contrast, for example, with inbred strains of laboratory mice. Such genetic variability could have a substantial effect on disease susceptibility. Bird ic2 did show a severe progression of clinical signs, and clinical signs developed in 4 other cockatiels. This variability in the course of ABV infection and development of PDD is similar to the variability observed in natural cases of ABV infection (*33*).

ABV RNA and antibodies to ABV were detected in all inoculated birds, and titers of antibody against ABV steadily increased during the investigation period; however, these antibodies did not influence the outcome of clinical disease. Payne et al. (*28*) also reported on ABV-infected cockatiels in which antibodies to ABV did not influence the outcome of clinical disease. Narayan et al. (*27*) similarly reported that in mammals with Borna disease, antibodies against ABV do not exhibit protective properties and seem not to play a role in immunopathogenesis. Thus, detection of antibodies to ABV does not indicate antiviral immunity; on the contrary, they indicate a resolved or ongoing ABV infection with possible risk for the development of PDD. The relevance of these findings should be considered in the diagnosis of possible ABV infections.

We detected ABV RNA considerably earlier in group ic than in group iv. This finding agrees with results of studies in which rats were experimentally infected with BDV and in which the IC route was shown to be the most efficient route for reproducing the disease; however, it was never possible in those studies to infect rats with BDV by iv inoculation (*27**,**34*). Therefore, the successful infection of cockatiels with ABV by IV inoculation represents a notable difference between ABV and BDV infection. This difference is highlighted by similarities in the postinoculation timing of seroconversion and the appearance of clinical signs in bird groups iv and ic. Gastrointestinal as well as neurologic signs could be noted in some birds of both groups. Moreover, we detected infectious virus and ABV RNA in all organs of all infected birds, whereas BDV exhibits strict neurotropism in immune-competent mammals, and spread of the virus to peripheral organs is only possible in immune-incompetent mammals (*30**,**35*). Whether this is also the case for ABV infection remains unclear. However, in our studies, ABV RNA was constantly present at the highest levels in the central nervous system and gastrointestinal tract, indicating that ABV also has an affinity for central and peripheral nervous tissue, as previously described (*13**,**18**,**20**,**21**,**36*). Additional investigations are warranted to resolve this issue.

Antibodies to ABV did not develop in the sentinel bird (se1) during the investigation period; however, increasing amounts of ABV RNA were detected in swab samples from the bird beginning 76 dpi. After se1 was euthanized, ABV RNA was amplified from the bird’s skin with feathers but not from other organs. The presence of ABV RNA in skin with feathers might be a result of contamination by virus that was constantly shed from other birds in the same cage. In contrast, se1 did show lymphoplasmacytic infiltrates typical for PDD in brain, heart, intestine, or liver. These findings indicate that bird se1 reacted differently than the experimentally infected birds, and it may have been more efficient than the experimental cohort in combatting the infection. This might be a result of infection by a different route, such as the oral or intranasal route. Additional investigations are needed to characterize the effects of different infection routes on the outcome of ABV infection and the development of PDD. It is already known from BDV infection of rats that the route of infection determines the severity of disease and that an early up-regulation of BDV-specific CD4 T cells can efficiently protect against infection by the virus (*37*).

In summary, the experimental infection of cockatiels in this study provides reliable evidence that ABV can induce a persistent infection by various routes and lead to disease patterns similar to those in natural infection. Moreover, the etiologic role of ABV for the development of PDD was further confirmed in an adequately sized cohort of cockatiels. Our detailed investigation of clinical signs, seroconversion, histopathologic lesions, and various viral parameters allowed us to document essential data on the course and clinical outcome of ABV infections and on the similarities and differences between ABV and BDV infections. This will serve as a basis for further investigations on the underlying pathogenesis and the main contributing virus and host factors in ABV infection. It remains to be determined whether immunopathologic mechanisms that are based on a T cell–mediated immune reaction, as known for BDV infection, play a role in ABV infection and the development of PDD. Findings in the present study add to our understanding of the pathogenesis of ABV infection and will facilitate interpretation of clinical findings. Antibodies to ABV do not indicate immunity; instead, they point toward a resolved or ongoing ABV infection and a possible risk for the development of PDD.
